# Elevated extracellular particle concentration in plasma predicts in-hospital mortality after severe trauma

**DOI:** 10.3389/fimmu.2024.1390380

**Published:** 2024-06-12

**Authors:** Nils Becker, Niklas Franz, Akiko Eguchi, Alessa Wagner, Ramona Sturm, Helen Rinderknecht, Yoshinao Kobayashi, Motoh Iwasa, Birte Weber, Ingo Marzi, Borna Relja

**Affiliations:** ^1^ Department of Trauma, Hand, Plastic and Reconstructive Surgery, Translational and Experimental Trauma Research, Ulm University Medical Center, Ulm, Germany; ^2^ Department of Trauma, Hand and Reconstructive Surgery, Goethe University Frankfurt, Frankfurt, Germany; ^3^ Department of Gastroenterology and Hepatology, Mie University Graduate School of Medicine, Tsu, Japan

**Keywords:** polytrauma, biomarker, death, extracellular vesicles, prediction

## Abstract

**Background:**

Extracellular particles (EPs), particularly extracellular vesicles, play a crucial role in regulating various pathological mechanisms, including immune dysregulations post-trauma. Their distinctive expression of cell-specific markers and regulatory cargo such as cytokines or micro-ribonucleic acid suggests their potential as early biomarkers for organ-specific damage and for identifying patients at risk for complications and mortality. Given the critical need for reliable and easily assessable makers to identify at-risk patients and guide therapeutic decisions, we evaluated the early diagnostic value of circulating EPs regarding outcomes in severely injured multiple-trauma patients.

**Methods:**

Plasma samples were collected from 133 severely injured trauma patients (Injury Severity Score (ISS) ≥16) immediately upon arrival at the emergency department (ED). Patients were categorized into survivors and non-survivors. Injury characteristics and outcomes related to sepsis, pneumonia, or early (<1 day after admission) and late mortality were assessed. Circulating EPs, cytokine profiles, and blood counts of platelets and leukocytes were determined. Receiver operating characteristic analyses were conducted.

**Results:**

Despite no significant differences in injury pattern or severity, non-survivors exhibited significantly elevated counts of circulating EPs compared to survivors. The optimal cut-off for EPs <200 nm indicating non-survivors was 17380/µl plasma, with a sensitivity of 77% and a specificity of 61% in predicting in-hospital mortality. Later non-survivors received significantly higher numbers of units of packed red blood cells [8.54 ± 5.45 vs. 1.29 ± 0.36 units], had higher serum lactate [38.00 ± 7.51 vs. 26.98 ± 1.58 mg/dL], significantly lower platelet counts [181.30 ± 18.06 vs. 213.60 ± 5.85 *10³/µL] and lower heart rates [74.50 ± 4.93 vs. 90.18 ± 2.06 beats/minute] upon arrival at the ED compared to survivors.

**Conclusion:**

Our results demonstrate the high diagnostic potential of elevated concentrations of circulating EPs <200 nm for identifying patients at risk of mortality after severe trauma. This parameter shows comparable sensitivity to established clinical predictors. Early evaluation of EPs concentration could complement assessment markers in guiding early therapeutic decisions.

## 1 Introduction

Individuals experiencing severe multiple trauma exhibit an increased likelihood of developing complications, which may arise hours or days after surviving the initial trauma (1). While early mortality is mainly influenced by the severity of the initial trauma, delayed mortality correlates with the development of complications (2). Although the impact of severe head injuries on trauma-related mortality compared to conditions like sepsis and multiple-organ failure has increased, infections remain the most commonly observed complication among severely multiple traumatized individuals (2, 3).

Recent studies have emphasized the role of circulating trauma-induced particles, particularly extracellular vesicles (EVs), in the development of complications. EVs, a heterogeneous group of small, cell-derived lipid-bilayer particles, carry specific cargo such as proteins, lipids and diverse forms of ribonucleic acid or deoxyribonucleic acid (4), reflecting their cellular source (5). In severe trauma, elevated levels of EVs have been observed independently of total leukocyte and platelet counts (5, 6). Additionally, differences in origin-specific EVs correlated with injury severity (7) and post-trauma outcomes (6). Thus, several studies focused on EVs` influence on coagulation and immune responses, while lower levels of procoagulant EVs have been shown to correlate with elevated post-traumatic mortality (6). Especially their immunomodulatory properties can promote various severe post-traumatic immune dysregulations, such as pneumonia (8) or sepsis (9). Due to their specific cellular origin, regulatory capabilities, and correlation with clinical outcome parameters, EVs show a high potential as biomarkers for organ-specific damage and early indicators and promotors of complication development. Furthermore, understanding their role in intercellular communication, homeostasis, and dysregulation has led to considering EVs as promising therapeutic targets in traumatized patients (5, 10).

However, despite the increasing literature on EVs, basic information such as the correlation between total and injury-severity-independent EV numbers and outcome remains elusive (5). Also, standardization of EV research is challenging due to variations in isolation and characterization methods, significantly influencing the integrity and bioactivity of EVs (11). Additionally, different nomenclatures have been used to discriminate fractions of EVs with distinct characteristics, including size- and origin-based terms. In an attempt to unify the nomenclature and characterization guidelines, which are crucial given the increasing literature on EVs, the Minimal Information for Studies of Extracellular Vesicles (MISEV) guidelines from 2023 (12) aim to improve the accuracy and comparability of EV studies. These guidelines include the analysis of different markers (both surface and cargo) from several categories, thereby making the definite analysis of EVs more accurate and comparable. When no analysis of characterization makers is performed, the term extracellular particles (EPs) was suggested (12). Despite the difficulties regarding an unified classification and isolation standard, the high potential of circulating EPs as regulators of the systemic response to trauma has been highlighted repeatedly, regardless of the different isolation and characterization methods used (13).

This study aims to evaluate the diagnostic value of the readily accessible size-dependent concentration of extracellular particles in the plasma of multiple traumatized patients compared to clinical markers. Early recognition of patients at risk of mortality and complications is critical for timely therapeutic decisions. Therefore, we focused on a multifactorial approach incorporating quick size differentiation of extracellular particles without their isolation alongside standard clinical and early laboratory parameters, which could be easily implemented in emergency and point-of-care diagnostics.

## 2 Materials and methods

### 2.1 Ethics

Patients were included at the University Hospital of the Goethe University Frankfurt with the approval of the institutional ethics committee (number of the ethical approval: 312/10) in accordance with the Declaration of Helsinki and following STROBE-guidelines (14). According to the ethical standards written informed consent was obtained from all enrolled subjects. Furthermore, all enrolled subjects signed the informed consent forms themselves or informed consent was obtained from the nominated legally authorized representative consented on the behalf of participants as approved by the ethical committee.

### 2.2 Inclusion and exclusion criteria

One hundred and thirty-three patients with acute trauma presenting to the ED with an Injury Severity Score (ISS) ≥16 between 18 and 80 years of age were included. All patients with known pre-existing immunosuppressive and anti-coagulant medication, any immunological disorders, burns, concomitant acute myocardial infarction, or thromboembolic events were excluded. Patients who died immediately after admission were excluded. For blood withdrawal, patients were included prospectively, however, the assessment of extracellular particles was performed retrospectively according to the availability of patients` material (500 μl plasma each) for this study and resulted in 133 patients that were analyzed.

### 2.3 Initial patient assessment, treatment and clinical data acquisition

The patients were treated upon admission according to the Advanced Trauma Life Support (ATLS) standards and the polytrauma guidelines (15, 16). Upon arrival at the ED, vital signs were recorded. Each injury was assigned the corresponding Abbreviated Injury Scale (AIS) score at discharge by a trained physician, which ranges from 1 (least severe injury) to 6 (unsurvivable) (17). The AIS score is allocated to one of six body regions, head/neck, face, chest, abdomen, extremities and external (17). The Injury Severity Score (ISS) is the sum of the squares of the highest AIS code in each of the three most severely injured body regions (18). Patients were grouped as “survivor” or “non-survivor” according to their clinical course. Furthermore, matched-pair analysis according to the ISS (± 2), age (± 5 years of age), and sex was conducted in 13 pairs. Blood samples were obtained, and parameters were determined as described below.

Hemodynamically unstable patients received immediate surgery when indicated, while stable patients underwent whole-body computed tomography. Upon arrival at the ED, the following clinical parameters were assessed: blood pressure, heart and respiratory rate, body temperature, and mechanism of injury. Routine blood gas analysis (including pH and lactate level) and coagulation parameters (thromboplastin time, TPT; partial TPT, PTT; international normalized ration, INR; fibrinogen, and platelets, PLT) were assessed upon admission to the ED. The numbers of packed red blood cell (PRBC) units and fresh frozen plasma (FFP) transfused within the ED, the first 24 hours, and during the further clinical course were recorded. Furthermore, leukocyte counts, C-reactive protein (CRP) and cytokines were determined. Blood counts (leukocytes and platelets) were obtained by standard clinical methods using the Sysmex XE-2100 automated blood cell counter (Sysmex Europe GmbH, Norderstedt, Germany). The diagnosis of sepsis was assessed by the revised definition criteria according to Sepsis-3 criteria (19, 20). Septic shock was clinically identified by a vasopressor requirement to maintain a mean arterial pressure of 65 mm Hg or greater and a serum lactate level greater than 2 mmol/L (>18 mg/dL) after adequate fluid resuscitation (20). Pneumonia development during the post-traumatic clinical course was defined by radiologic, clinical, and bacteriologic findings with the presence of new pulmonary infiltrates on chest X-ray and at least one of the following criteria: positive blood culture, bronchial alveolar lavage, and/or sputum culture. Medical records were analyzed regarding the length of in-hospital stay, length of stay in the intensive care unit (ICU), and early (within 24 hours) as well as late occurring in-hospital mortality (from post-trauma day 1 until observational day 10).

### 2.4 Blood processing and analysis

Blood samples were obtained from severely injured trauma patients as early as possible upon admission to the ED for routine diagnostics and laboratory investigations, as described previously (21). Blood samples were collected in pre-chilled ethylenediaminetetraacetic acid (EDTA) tubes (BD vacutainer, Becton Dickinson Diagnostics, Aalst, Belgium) and kept on ice. The samples were then centrifuged at 2000 × g for 15 min at 4°C, and the supernatant was stored at -80°C until analysis. Cytokine concentrations were measured using an IL-6 and IL-10 Eli-pair ELISA-Assay (Diaclone, Hoelzel Diagnostica, Cologne, Germany) according to the manufacturer’s instructions.

### 2.5 Analysis of extracellular particles

Circulating EPs in plasma samples from traumatized patients were analyzed as previously described (21–23). Briefly, plasma was centrifuged at 2000 × *g* for 15 min at 4°C to eliminate any aggregations. Then, EPs were stained with 4 μg/mL of calcein-AM (Invitrogen, San Diego, CA) for 30 minutes at room temperature in the dark. Using 2.5 μm UV-conjugated Alignflow alignment beads (Life Technologies) as the size standards for flow cytometry (BD Canto II; BD Biosciences, San Jose, CA), the total number of EPs was determined (**Supplementary Figure 1**). All positively stained particles were counted as all events, while particles with a size <200 nm were specifically gated and analyzed as EPs <200 nm. The data were analyzed using FlowJo software (TreeStar, Ashland, OR).

For the validation of the measurement approach, the gating strategy was confirmed using several differently sized beads [Spherotech nano fluorescent particle size standard kit (Spherotech, Lake Forest, IL) and FluoSpheres biotin-labeled 0.04 μm yellow-green (Life Technologies)]. We validated the intraluminal signal of Calcein by adding 0.3% Triton X-100 (BioXtra, Sigma-Aldrich, St. Louis, MO) to Calcein-stained EDTA-plasma of healthy volunteers and vortexing for 30 seconds. We observed a complete loss of Calcein-AM positive signals in the flowcytometric analysis (data not shown). This is in line with the results of previously described validation approaches (24). A simplified flowchart of the EP analysis is added in the **Supplementary Material** (**Supplementary Figure 1**).

### 2.6 Statistical analysis

The unpaired non-parametric Mann Whitney U test was applied to assess the differences among the groups. Chi-square test was applied for the analyses of proportions. Receiver–operator curves (ROC) were generated to analyze the optimal cutoff levels. Matched-pair analysis (n=26) according to the ISS, age, and sex was performed due to significant difference of age between the groups. Both groups were matched according to the ISS (± 2), age (± 5 years of age), and sex, to achieve a reliable statistical comparability, followed by the reevaluation of the data set. Thirteen pairs were included. Data are presented as the mean ± standard error of the mean (sem) unless otherwise stated. A p-value <0.05 was considered statistically significant. GraphPad Prism 6.0 software (GraphPad Software Inc. San Diego, CA) was used to perform the statistical analysis.

## 3 Results

### 3.1 Study cohort

A total of 133 patients admitted to the emergency department with severe trauma met the inclusion criteria and were considered for our study cohort (**Table 1**). The mean time between the injury and the blood sampling in the ED was 66.50 ± 3.13 minutes. Thirteen patients were included in the non-survivor group, while 120 patients were evaluated in the survivor group. All patients were substantially injured with a median ISS of 25 (IQR: 18–34). There was no significant difference between survivors and non-survivors in terms of the overall injury severity. Detailed analyses of the injured body regions have revealed no significant differences in individual AIS compartments (head, chest, abdomen, extremity) either (**Table 1**). The mean age of all patients was 44.53 ± 1.53 years of age (non-survivors vs. survivors: 54.33 ± 4.38 vs. 43.52 ± 1.59, p <0.05).

**Table 1:** An overview of patients and injury characteristics during the presentation to the emergency department (ED).

trauma severity and mechanism	all patients (n = 133)	non-survivor (n = 13)	survivor (n = 120)	p <0.05 non-survivor *vs*. survivor
**ISS (25% and 75% percentile)**	25 (18 and 34)	25 (21 and 39)	24 (18 and 34)	n.s
mechanism of injury				
**falls, % (n)**	42.11%, (56)	53.85%, (7)	40.83%, (49)	n.s
**other, % (n)**	57.89%, (77)	46.15%, (6)	59.17%, (71)	
AIS ≥ 3				
**head, n, %**	65, 48.87%	9, 69.23%	56, 46.67%	n.s
**chest, n, %**	68, 51.13%	5, 38.46%	63, 52.50%	n.s
**abdomen, n, %**	20, 15.04%	1, 7.69%	19, 15.83%	n.s
**extremity, n, %**	30, 22.56%	2, 15.38%	28, 23.33%	n.s
**time to ED presentation (min)**	66.50 ± 3.13	66.00 ± 5.15	66.55 ± 3.40	n.s.

Three investigated groups are shown (all patients, non-survivors and survivors). Data are given as mean ± standard error of the mean or median (ISS) with 25% and 75% percentile, p <0.05. AIS, Abbreviated Injury Scale; ISS, Injury Severity Score; n.s., no significance.

Matched-pair analysis of thirteen pairs according to the ISS, age, and sex was performed due to significant difference of age between the groups. The ISS [25 (IQR 21–39) vs. 25 (IQR 21–39)], age (54.33 ± 4.38 vs. 58.54 ± 4.41 years of age) and sex were comparable, as were the mechanisms of injury, AIS scores and the time until admission to the ED (**Supplementary Table 1**).

### 3.2 Physiologic characteristics and laboratory parameters

The assessment of physiological and laboratory parameters showed that although there were no significant differences among mean systolic blood pressure (SBP), shock index, the respiratory rate or temperature, the survivors had a significantly increased heart rate compared to non-survivors (74.50 ± 4.93 vs. 90.18 ± 2.06, p <0.05, **Table 2**). The non-survivor group had a significantly increased need for PRBC transfusion in the ED (8.54 ± 5.45 vs. 1.29 ± 0.36, p <0.05, **Table 2**). There were no further significant differences in the reperfusion volume regarding neither the PRBC transfusion within 24 hours after injury or total transfusion rates during the ten observational study days. Similarly, there were no significant differences in the FFP volume that was applied at any time point (**Table 2**), while the FFP transfusions were significantly higher in the non-survivors group after matching. Although there were no significant differences regarding the coagulation parameters (TPT, PTT, INR, fibrinogen) between the two groups, significantly lower platelet counts were found in the non-survivor group compared to the survivor group (181.30 ± 18.06 vs. 213.60 ± 5.85, p <0.05, **Table 2**). Lactate was significantly higher in the non-survivor group compared to the survivor group (38.00 ± 7.51 vs. 26.98 ± 1.58, p <0.05, **Table 2**), which was also confirmed after matching. The levels of IL-6 and IL-10 were found to be higher in the survivor group compared to the non-survivor group; however, this difference was not significant (**Table 2**). This trend was observed after matching as well (**Supplementary Table 2**).

**Table 2:** Physiologic characteristics and laboratory parameters of the study population at admission.

physiological and laboratory measurements	all patients (n = 133)	non-survivor (n = 13)	survivor (n = 120)	p <0.05 non-survivor *vs*. survivor
**SBP, mm Hg**	132.00 ± 2.90	127.80 ± 10.17	132.50 ± 3.03	n.s.
**shock index (HR/SBP)**	0.74 ± 0.03	0.63 ± 0.06	0.75 ± 0.03	n.s.
**heart rate**	88.71 ± 1.96	74.50 ± 4.93	90.18 ± 2.06	***yes***
**breath rate**	14.24 ± 0.42	16.00 ± 2.29	14.03 ± 0.39	n.s.
**body temperature (°C)**	36.03 ± 0.09	35.99 ± 0.38	36.03 ± 0.09	n.s.
**PRBC transfusion ED (Units)**	1.86 ± 0.65	8.54 ± 5.45	1.29 ± 0.36	***yes***
**PRBC transfusion within 24 h (Units)**	4.71 ± 0.87	8.70 ± 6.88	4.26 ± 0.73	n.s.
**PRBC transfusion total (Units)**	7.04 ± 4.14	9.80 ± 6.84	6.89 ± 4.48	n.s.
**FFP transfusion within 24 h (Units)**	2.21 ± 0.72	8.00 ± 6.28	1.63 ± 0.48	n.s.
**FFP transfusion total (Units)**	2.33 ± 0.73	8.60 ± 6.90	1.78 ± 0.51	n.s.
**hemoglobin, g/dL**	12.73 ± 0.20	11.89 ± 0.64	12.82 ± 0.20	n.s.
**TPT (thromboplastin time), %**	85.61 ± 1.62	80.92 ± 5.21	86.13 ± 1.70	n.s.
**PTT (partial thromboplastin time), (sec)**	29.19 ± 0.52	31.42 ± 2.23	28.97 ± 0.53	n.s.
**INR**	1.154 ± 0.024	1.199 ± 0.067	1.149 ± 0.025	n.s.
**fibrinogen, mg/dL**	215.20 ± 6.40	196.90 ± 13.87	217.30 ± 6.95	n.s.
**PLT count, x 10^3^/µL**	210.40 ± 5.61	181.30 ± 18.06	213.60 ± 5.85	***yes***
**pH**	7.286 ± 0.010	7.265 ± 0.041	7.288 ± 0.010	n.s.
**lactate, mg/dL**	27.72 ± 1.58	38.00 ± 7.51	26.98 ± 1.58	***yes***
**leukocytes, U/nL**	12.86 ± 0.50	10.34 ± 0.88	13.13 ± 0.54	n.s.
**CRP, mg/dL**	0.33 ± 0.06	0.33 ± 0.10	0.33 ± 0.07	n.s.
**IL-6, pg/mL**	151.20 ± 21.45	80.46 ± 25.34	158.20 ± 23.35	n.s.
**IL-10, pg/mL**	97.68 ± 15.33	19.24 ± 5.03	105.00 ± 16.61	n.s.

Three investigated groups are shown (all patients, non-survivors and survivors). Data are given as mean ± standard error of the mean, p <0.05. CRP, C-Reactive Protein; ED, Emergency Department; FFP, Fresh Frozen Plasma; IL, Interleukin; INR, International Normalized Ratio; n.s., no significance; PLT, Platelets; PRBC, Packed Red Blood Cells; PTT, activated Partial Thromboplastin Time; SBP, Systolic Blood Pressure; TPT, Thromboplastin Time.

### 3.3 Outcome

The length of stay in the ICU was significantly longer in survivors compared to non-survivors (11.31 ± 1.18 vs. 4.62 ± 1.32 days, p <0.05, **Table 3**). A similar distribution was found in the total length of the stay in the hospital, demonstrating that survivors had significantly longer stay compared to non-survivors (24.21 ± 1.81 vs. 5.15 ± 1.22 days, p <0.05, **Table 3**). Subdividing the non-survivors in early and late occurring deaths showed a significant higher number of late mortality cases (23.08% vs. 76.92%, p <0.05, **Table 3**). There were no significant differences in terms of post-traumatic infectious complication pneumonia, sepsis or septic shock, however, a trend towards a higher rate of infectious complications in survivors was observed (**Table 3**). The results were comparable in regard to significant and not significant changes after matched-pair analyses (**Supplementary Table 3**).

**Table 3:** Outcome of the study population.

outcome	all patients (n = 133)	non-survivor (n = 13)	survivor (n = 120)	p <0.05 non-survivor *vs*. survivor
**length of ICU stay (days)**	10.64 ± 1.09	4.62 ± 1.32	11.31 ± 1.18	***yes***
**length of hospital stay (days)**	22.35 ± 1.71	5.15 ± 1.22	24.21 ± 1.81	***yes***
**early mortality (<1 day), % (n)**	2.26%, (3)	23.08%, (3)	–	*early vs. late* ***yes***
**later mortality (d1-10 days), % (n)**	7.52%, (10)	76.92%, (10)	–	
**pneumonia, % (n)**	28.57%, (38)	15.38%, (2)	30.00%, (36)	n.s.
**sepsis, % (n)**	18.05%, (24)	7.69%, (1)	19.17%, (23)	n.s.
**septic shock, % (n)**	5.26%, (7)	7.69%, (1)	5.00%, (6)	n.s.

Three investigated groups are shown (all patients, non-survivors and survivors). Data are given as mean ± standard error of the mean, p <0.05. d, day; ICU, Intensive Care Unit; n.s., no significance.

### 3.4 Enhanced numbers of extracellular particles in non-survivors predict mortality after trauma

**Figure 1:**
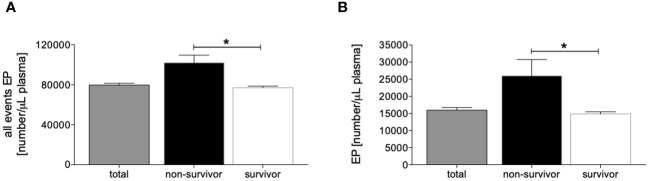
Distribution of measured circulating all events [number/µL plasma, **(A)**] and extracellular particle (EP) <200 nm size [number/µL plasma, **(B)**] in all patients (n=133), non-survivors (n=13) and survivors (n=120). Data are given as mean ± standard error of the mean, *: p <0.05 vs. indicated.

The assessment of all events and totally measured EPs in plasma samples from traumatized patients showed a significantly increased number of total events as well as EPs below 200 nm size in the non-survivor group compared to the survivor group (p <0.05, **Figures 1A, B**). Identical results were observed after matched-pair analyses (**Supplementary Figure 2**).

**Figure 2:**
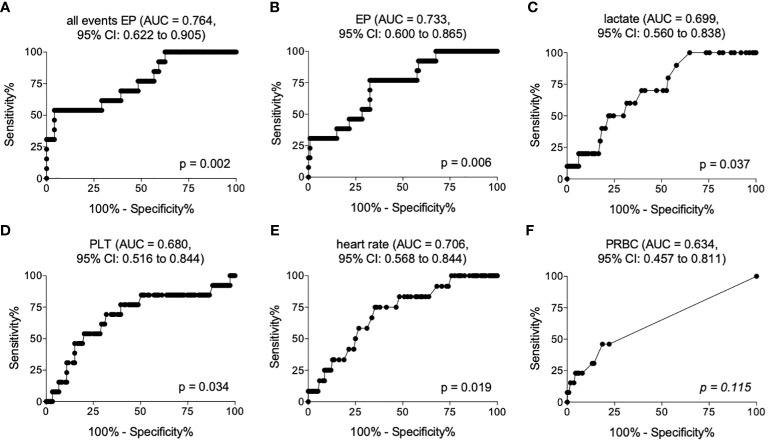
Receiver operating curves showing area under the curve (AUC) and the optimal cut-off values for all events **(A)**, EP below 200 nm size **(B)**, lactate **(C)**, platelet counts (PLT) **(D)**, heart rate **(E)** and amount of packed red blood cell transfusion (PRBC) **(F)** in the emergency department discriminating later non-survivors from survivors.

The ROC analysis for all detected events showed an optimal cut-off for distinguishing later survivors in the clinical course from non-survivors at 85059 number/µl plasma with 69.23% sensitivity (95% CI = 38.57% to 90.91%), 60.83% specificity (95% CI = 51.50% to 69.61%) and an AUC of 0.764 (95% CI = 0.622 to 0.905) (p <0.05, **Figure 2A** and **Table 4**).

**Table 4:** Cut-off values with corresponding sensitivity and specificity for distinguishing survivors from non-survivors after trauma.

parameter	cut-off value	sensitivity % (95% CI)	specificity % (95% CI)	AUC (95% CI)	p <0.05
**all events EP [number/μL plasma]**	85059	69.23 (38.57 to 90.91)	60.83 (51.50 to 69.61)	0.764 (0.622 to 0.905)	***yes***
**EP [number/μL plasma]**	17380	76.92 (46.19 to 94.96)	60.83 (59.05 to 76.81)	0.733 (0.600 to 0.865)	***yes***
**lactate [mg/dL]**	25.50	70 (34.75 to 93.33)	60.53 (50.94 to 69.55)	0.699 (0.560 to 0.838)	***yes***
**PLT count [x10^3^/μL]**	182.00	69.23 (38.57 to 90.91)	68.07 (58.90 to 76.31)	0.680 (0.516 to 0.844)	***yes***
**heart rate [beat/minute]**	81.50	75.00 (42.81 to 94.51)	64.66 (55.24 to 73.31)	0.706 (0.568 to 0.844)	***yes***

P <0.05. AUC, Area Under the Curve; CI, Confidence Interval; ED, Emergency Department; EP, Extracellular Particles; PLT, Platelets.

The ROC analysis for EPs depicted an optimal cut-off for mortality prediction at 17380 number/µl plasma with 76.92% sensitivity (95% CI = 46.19% to 94.96%), 60.83% specificity (95% CI = 59.05% to 76.81%) and an AUC of 0.733 (95% CI = 0.600 to 0.865) (p <0.05, **Figure 2B** and **Table 4**).

The ROC analysis for lactate showed an optimal cut-off for distinguishing later survivors in the clinical course from non-survivors at 25.50 mg/dL with 70% sensitivity (95% CI = 34.75% to 93.33%), 60.53% specificity (95% CI = 50.94% to 69.55%) and an AUC of 0.699 (95% CI = 0.560 to 0.838) (p <0.05, **Figure 2C** and **Table 4**).

The ROC analysis of PLT demonstrated an optimal cut-off for mortality prediction at 182*10³/µL with 69.23% sensitivity (95% CI = 38.57% to 90.91%), 68.97% specificity (95% CI = 58.90 to 76.31%) and an AUC of 0.680 (95% CI = 0.516 to 0.844) (p <0.05, **Figure 2D** and **Table 4**).

The ROC analysis of the heart rate showed an optimal cut-off for distinguishing later survivors in the clinical course from non-survivors at 81.50 beats/minute with 75.00% sensitivity (95% CI = 42.81% to 94.51%), 64.66% specificity (95% CI = 55.24% to 73.31%) and an AUC of 0.706 (95% CI = 0.568 to 0.844) (p <0.05, **Figure 2E** and **Table 4**).

Although there were significant differences in the PRBC transfusion rates, the PRBC did not provide a significant predictive ability for the assessment of post-traumatic mortality (**Figure 2F**).

## 4 Discussion

In this study, the diagnostic utility of early clinical markers and systemic concentrations of extracellular particles, including both the overall number of extracellular particles and those size characteristic of extracellular vesicles (<200 nm), in predicting adverse outcomes following multiple trauma was investigated. Significant differences in lactate levels, total platelet counts, heart rate, and circulating numbers of both total and size-dependent EPs between survivors and non-survivors were found. Specifically, we observed that mortality rates were indicated by elevated EP levels. These findings suggest that assessing circulating EPs could aid in early risk stratification and therapeutic decision-making in multiple traumatized patients.

A cohort of 133 patients with a median ISS of 25 was examined, revealing an overall mortality rate of 10.2%. This aligns with other studies, albeit at the lower range (3). Notably, mortality rates in clinical studies are significantly influenced by inclusion criteria. The exclusion of patients with anticoagulant medication, which is more prevalent among older patients (25), as well as patients who died immediately after admission, might have contributed to the relatively low mortality rates observed in our study, given that age is a known risk factor for trauma-associated mortality (2, 26). This is also indicated by significantly higher age among non-survivors compared with survivors in our study; however, the results remain significant after matching the non-survivors with survivors according to the injury severity, sex and age (**Supplementary Figure 2**).

Regarding the timing of trauma-associated mortality, 23% of deaths occurred within 24 hours of admission, while 77% occurred beyond this timeframe (**Table 3**). As several studies show a decrease in trauma-associated mortality over time (2, 27), our data underlines the high relevance of delayed trauma-associated mortality. While early mortality is commonly related to the initial injury severity, delayed mortality is more frequently associated with the development of complications such as early (28) or late multi-organ failure and infections (2, 29). In our analysis, rates of pneumonia and sepsis did not significantly differ between survivors and non-survivors (**Table 3**), although there was a trend toward lower rates in non-survivors. This is consistent with previous findings in different study populations (2). Our study did not discriminate between different time points for later mortality, potentially diminishing the attributed influence of complication development, as infections such as pneumonia or sepsis typically take several days to develop and contribute to trauma-associated mortality (29). However, looking at the statistics between the early mortality group (n = 3) and the late mortality group (n = 10), in the early cohort significantly enhanced lactate levels were found (65.00 ± 16.64 versus 26.43 ± 2.62, p = 0.0167). Traumatic brain injury was not significantly enhanced in the early mortality groups compared to the late mortality group (AIS ≥3 head (n, %): 1, 33.33% versus 8, 80.00%, no significance). However, in the early mortality group apparently the injuries of extremities (AIS ≥3 extremity (n, %): 2, 66.67% versus 0, 0%, p = 0.0050) and the probability of massive blood loss may constitute as the cause of early death. No differences in the ISS (ISS (25% and 75% percentile): 50 (45 and 50) in the early mortality cohort versus 25 (18 and 25) in the late mortality cohort), AIS for abdomen or chest were found. Yet, the study design which excluded patients who died immediately upon admission hides some limitations in regard to the early mortality after trauma defined as patients who died within 24 hours after admission. Also, no further specific data on the specific causes of death for the patient cohort in the current study were recorded. Notably the group size in the early mortality groups must be interpreted carefully, since due to three patients being included no reliable statistical credibility is expected. In addition, the “Do not resuscitate” forms were not considered in the evaluation, which is a subject to further larger future studies.

While the injury severity was comparable to other studies (30) and did not different between survivors compared with non-survivors, no significant differences in rates of severely injured body-regions were observed (**Table 2**). The significance of head injuries has been emphasized in recent global meta-analysis, which highlighted the elevated mortality associated with severe head injuries (3). Given that the primary traumatic brain damage cannot be directly targeted therapeutically, acute treatment strategies focus on the prevention of ischemia and edema-associated secondary injuries (31, 32). Additionally, traumatic brain injuries can lead to changes in circulating factors such as miR-142–3p (33) and cytokine profiles (34), potentially contributing to dysregulated inflammatory responses, complications like pneumonia (35) and adverse outcomes. Recent studies have shown that these circulating immune-modulating markers are transported in extracellular particles after multiple-trauma, with distinct cytokine levels in vesicles exhibiting different dynamics compared to free cytokines (36), underlining the lasting regulatory potential of extracellular particles. In our study, non-survivors, who showed a trend toward elevated rates of severe head injuries, exhibited increased levels of extracellular particles compared to survivors. This suggests a potential contribution of traumatic brain injuries to the elevation of extracellular particles. It was shown that the injury pattern affects the surface marker expression on exosomes in multiple-trauma patients. In this context TBI was associated with elevated rates of CD62p^+^ exosomes, which are released by endothelial cells, platelets and megakaryocytes (37). Little is known about the role of CD62p^+^ cells and exosomes in TBI, however in a different setting a correlation between CD62p^+^ platelets and exosomes to disrupted endothelial cell integrity has been described (38). This could influence the recovery after TBI and potentially promote posttraumatic cerebral edema. Furthermore, microglial-derived microparticles that are released after TBI can induce neuroinflammation (39), while these pro-inflammatory influences could also induce systemic inflammatory changes. Aside from the exosome profile in TBI, other injury patterns have shown distinct changes in the exosome profile, while no reliable marker for single organ injuries has been reported yet, despite promising results (13).

We observed, that non-survivors received significantly more transfusions within the first 24 hours after admission, and elevated concentrations of EPs have shown to influence coagulation, potentially leading to disseminated intravascular coagulation and increased blood loss (40–42). However, elevated levels of EPs may also result from increased transfusion rates, as stored packed red blood cells contain high concentrations of red blood cell-derived extracellular particles (43), which can induce inflammatory responses (44). Due to our study-design with blood-sampling in the emergency department just after the admission, only very early transfusions in hemodynamic-instable patients before blood sampling could have influenced the EP number. Regardless of the early EP assessment before transfusion, further studies are needed to evaluate the influence of transfusion-derived extracellular particles in multiple traumatized patients, regarding the correlation between transfusion rates and early elevated levels of extracellular particles in non-survivors, also considering storage conditions of packed red blood cells, as they influence the concentration of EVs (44). This is especially relevant, if the dynamic of EPs in the clinical course is assessed.

Elevated transfusion rates have been correlated with adverse outcomes in multiple traumatized patients and are used as prognostic markers in scoring systems as the PolyTrauma Grading Score (PTGS) (45). Additional significant factors correlated with mortality in the PTGS include decreased systolic blood pressure and lower platelet counts, which align with our findings (**Table 4**) (45). Interestingly, systolic blood pressure and lactate levels have recently been evaluated as important markers for distinguishing clinically stable, unstable, or borderline multiple traumatized patients (46), with similar cut-off values as in our study population (**Table 4**). Lactate levels have been associated with early post-traumatic mortality, although their diagnostic value may be compromised by confounding factors such as alcohol intoxication (47).

In our study, the diagnostic value of identifying multiple traumatized patients at-risk based on the total circulating extracellular particle count and extracellular particles within the typical size range of extracellular vesicles (<200 nm) is comparable in terms of specificity and sensitivity to established factors (**Table 4**). These parameters can be easily and quickly assessed in an acute setting and integrated into early decision-making processes regarding therapy (46).

In our correlation analysis (**Supplementary Table 4**) we were able to show, that the total EP number significantly correlated with age (r = 0.18, p = 0.0436), leukocyte count (r = 0.17, p = 0.0467), systolic blood pressure (r = 0.22, p = 0.0139), shock index (r = -0.24, p = 0.008), and heart rate (r = -0.18, p= 0.0366).

Age-dependent differences in the cargo and concentration of extracellular vesicles have been described before. While some authors described decreased rates of EVs in aged healthy controls comparted to a younger cohort, other authors have not been able to validate significant age-dependent differences (48). The correlation of age with EP numbers in our cohort could indicate an age-specific elevation of EPs in a multiple-trauma setting. However, in our matched-pair analysis elevated EPs numbers remained a significant indicator for mortality.

Hematopoietic cells are main producers of EVs in the circulation (49), thus elevations in total EP numbers might be associated with a higher production of EPs by increased cell numbers. Interestingly, leukocytes and platelets in non-survivors of our cohort showed a trend to be decreased compared to survivors, despite elevated rates of EPs (**Table 2**). Therefore, the elevation of EPs in non-survivors does not derive from an elevated number of leukocytes. The elevated number of EPs might however derive from a higher secretion by activated cells, as stimulation of leukocytes can increase their EV release (50) and could indicate an immune dysregulation. In this context the correlation between EP numbers and systolic blood pressure and the negative correlation between EP numbers and shock index or heart rate can lead to the conclusion, that elevated rates of EPs correlate with stable patients, despite their elevation in non-survivors. Potentially, elevated levels of EPs indicate a dysregulated state at a very early level or even promote dysregulations due to their high regulatory capacity.

Regarding the critical immune response of severely injured patients, highly susceptible to develop dysregulations, operations and interventions have to be planned carefully and always in consideration of the current clinical situation (46). Our findings might indicate patients with elevated EP concentrations to benefit from a minimal-invasive surgical approach, as trends towards lower IL-6 levels in non-survivors could indicate early immune-dysregulation (**Table 2**). However, before implementation in clinical algorithms and existing scoring systems, we endorse the validation in prospective studies, while our results highlight EPs as promising target. Given the heterogenous population of multiple-trauma patients, the interpretation of EP numbers in combination with existing assessment markers might provide the highest specificity. Despite challenges in defining extracellular vesicle according to MISEVs guidelines (12), our approach facilitates a rapid assessment. However, correlations between size-dependent quantitative assessments and validation of markers for extracellular vesicles have been described in different models, supporting our findings (23). This is underscored by the growing body of evidence regarding the importance of extracellular vesicles in individuals experiencing multiple trauma (5, 7). Extracellular vesicles, characterized by their lipid-coated structure and expression of various cellular intra-vesicular and surface markers, depending on their cellular origin, hold significant promise as biomarkers for organ- or injury-specific damage (9, 13, 51–53). Interestingly, in contrast to our findings, Matijevic et al. reported decreased levels of microvesicles in non-survivors, highlighting the importance of clear definitions of sizes (54) and consideration of study population characteristics. While Matijevic et al. focused on particles sized between 500–1000 nm, our study differentiated particles into sizes less than 200 nm and greater than 200 nm. Additionally, variations in pre-analysis sample handling protocols, such as inclusion criteria (severe bleeding), and significant differences in injury severity between survivors (ISS 29) and non-survivors (ISS 41) (54) as compared to our study, can substantially impact the outcomes of analyses. The cut-off of 200 nm in our study was chosen to include and discriminate small EVs (<200 nm) including the biggest fractions of exosomes and larger fractions (>200 nm) (4), as recommended by the current MISEV guidelines. The size differentiation can provide information about the cargo of extracellular particles (55), although we are emphasizing that clear nomenclature demands the validation with extracellular versicle markers (12).

Despite these challenges, the correlation of cell-specific markers such as increased levels of platelet-derived and endothelia-derived microvesicles with injury severity (7) or distinct changes in extracellular vesicle profiles correlating with elevated mortality (6) makes extracellular vesicles a promising target for novel therapies. Efforts to standardize and accelerate extracellular vesicle isolation and characterization methods are essential for obtaining valid results. Our findings underline the promising diagnostic value of circulating extracellular particles, independent of isolation and injury severity, encouraging further investigation into their role in trauma-associated complications and mortality.

In conclusion, an elevated concentration of EPs <200 nm in the plasma of severely injured patients upon arrival in the emergency department is a significant indicator for trauma-induced mortality. The sensitivity and specificity are comparable to established clinical predictors. Thus, the assessment of the easily quantifiable EP concentrations in plasma could contribute as a key parameter in recognizing patients at risk for an adverse outcome. Considering their regulatory capacity and correlation to mortality after trauma, further effort in the characterization and evaluation of the therapeutic potential of EPs, including extracellular vesicles, is promising.

## Data availability statement

The raw data supporting the conclusions of this article will be made available by the authors, without undue reservation.

## Ethics statement

This study was performed at the University Hospitals of the Goethe-University Frankfurt with institutional ethics committee approval (312/10), and the University of Ulm, in accordance with the Declaration of Helsinki and following the Strengthening the Reporting of Observational studies in Epidemiology (STROBE)-guideline.

## Author contributions

NB: Data curation, Investigation, Visualization, Writing – original draft. NF: Conceptualization, Data curation, Investigation, Methodology, Visualization, Writing – original draft. AE: Conceptualization, Investigation, Methodology, Project administration, Supervision, Writing – review & editing. AW: Writing – review & editing. RS: Writing – review & editing. HR: Writing – review & editing. YK: Writing – review & editing. MI: Writing – review & editing. BW: Writing – review & editing. IM: Writing – review & editing. BR: Conceptualization, Data curation, Formal Analysis, Investigation, Methodology, Project administration, Supervision, Visualization, Writing – original draft.

## Supplementary material

Supplementary Figure 1 Flowchart of the used methodology to assess the extracellular particle analysis. EDTA, Ethylenediaminetetraacetic acid; EPs, extracellular particles. Created with *Biorender.com.*

Supplementary Figure 2 Distribution of measured circulating all events (number/µL plasma, **A**) and EP <200 nm size (number/µL plasma, **B**) in the matched population containing all matched patients (n=26), non-survivors (n=13) and survivors (n=13). Data are given as mean ± standard error of the mean, *: p <0.05 vs. indicated.

Supplementary Table 1 An overview of patients and injury characteristics and time to the presentation to the emergency department (ED) in the matched-pair population (n=26). Three investigated groups are shown (all matched patients, non-survivors (n=13) and survivors(n=13)). Data are given as mean ± standard error of the mean or median (ISS) with 25% and 75% percentile, p <0.05. AIS, Abbreviated Injury Scale; ISS, Injury Severity Score; n.s., no significance.

Supplementary Table 2 Physiologic characteristics and laboratory parameters of the matched-pair population at admission. Three investigated groups are shown [all matched patients (n=26), non-survivors (n=13) and survivors (n=13)]. Data are given as mean ± standard error of the mean, p <0.05. CRP, C-Reactive Protein; ED, Emergency Department; FFP, Fresh Frozen Plasma; IL, Interleukin; INR, International Normalized Ratio; n.s., no significance; PLT, Platelets; PRBC, Packed Red Blood Cells; PTT, activated Partial Thromboplastin Time; SBP, Systolic Blood Pressure; TPT, Thromboplastin Time.

Supplementary Table 3 Outcome of the matched-pair population (n=26). Three investigated groups are shown [all matched patients (n=26), non-survivors (n=13) and survivors (n=13)]. Data are given as mean ± standard error of the mean, p <0.05. d, day; ICU, Intensive Care Unit; n.s., no significance.

Supplementary Table 4 Correlation analysis and spearman r values between physiological and laboratory parameters and total extracellular particle (EP) numbers at the emergency department. CRP, C-Reactive Protein; FFP, Fresh Frozen Plasma; INR, International Normalized Ratio; PLT, Platelets; PRBC, Packed Red Blood Cells; SBP, Systolic Blood Pressure.

## References

[1] Pape HC Halvachizadeh S Leenen L Velmahos GD Buckley R Giannoudis PV. Timing of major fracture care in polytrauma patients - An update on principles, parameters and strategies for 2020. Injury. (2019) 50:1656–70. doi: 10.1016/j.injury.2019.09.021 31558277

[2] Becker N Hammen A Bläsius F Weber C Hildebrand F Horst K. Effect of injury patterns on the development of complications and trauma-induced mortality in patients suffering multiple trauma. J Clin Med. (2023) 12:5111. doi: 10.3390/jcm12155111 37568511 PMC10420136

[3] van Breugel JMM Niemeyer MJS Houwert RM Groenwold RHH Leenen LPH van Wessem KJP. Global changes in mortality rates in polytrauma patients admitted to the ICU-a systematic review. World J Emerg Surg. (2020) 15:55. doi: 10.1186/s13017-020-00330-3 32998744 PMC7526208

[4] van Niel G D'Angelo G Raposo G. Shedding light on the cell biology of extracellular vesicles. Nat Rev Mol Cell Biol. (2018) 19:213–28. doi: 10.1038/nrm.2017.125 29339798

[5] Alsaadi N Srinivasan AJ Seshadri A Shiel M Neal MD Scott MJ. The emerging therapeutic potential of extracellular vesicles in trauma. J Leukoc Biol. (2022) 111:93–111. doi: 10.1002/JLB.3MIR0621-298R 34533241 PMC9169334

[6] Curry N Raja A Beavis J Stanworth S Harrison P. Levels of procoagulant microvesicles are elevated after traumatic injury and platelet microvesicles are negatively correlated with mortality. J Extracell Vesicles. (2014) 3:25625. doi: 10.3402/jev.v3.25625 26077419 PMC4216813

[7] Frohlich M Schafer N Caspers M Böhm JK Stürmer EK Bouillon B. Temporal phenotyping of circulating microparticles after trauma: a prospective cohort study. Scand J Trauma Resusc Emerg Med. (2018) 26:33. doi: 10.1186/s13049-018-0499-9 29703240 PMC5921785

[8] Seibold T Schonfelder J Weeber F Lechel A Armacki M Waldenmaier M. Small extracellular vesicles propagate the inflammatory response after trauma. Adv Sci (Weinh). (2021) 8:e2102381. doi: 10.1002/advs.202102381 34713625 PMC8693079

[9] Weber B Henrich D Hildebrand F Marzi I Leppik L. The roles of extracellular vesicles in sepsis and systemic inflammatory response syndrome. Shock. (2023) 59:161–72. doi: 10.1097/SHK.0000000000002010 PMC9940838 36730865

[10] Han Y Zhu Y Almuntashiri S Wang X Somanath PR Owen CA. Extracellular vesicle-encapsulated CC16 as novel nanotherapeutics for treatment of acute lung injury. Mol Ther. (2023) 31:1346–64. doi: 10.1016/j.ymthe.2023.01.009 PMC10188639 36635966

[11] Monguio-Tortajada M Galvez-Monton C Bayes-Genis A Roura S Borras FE. Extracellular vesicle isolation methods: rising impact of size-exclusion chromatography. Cell Mol Life Sci. (2019) 76:2369–82. doi: 10.1007/s00018-019-03071-y PMC11105396 30891621

[12] Welsh JA Goberdhan DCI O`Driscoll L Buzas EL Blenkiron C Bussolati B. Minimal information for studies of extracellular vesicles (MISEV2023): From basic to advanced approaches [published correction appears in J Extracell Vesicles. 2024 May;13(5):e12451]. J Extracell Vesicles. 2024 ;13(2):e12404. doi: 10.1002/jev2.12404 PMC10850029 38326288

[13] Weber B Franz N Marzi I Henrich D Leppik L. Extracellular vesicles as mediators and markers of acute organ injury: current concepts. Eur J Trauma Emerg Surg. (2022) 48:1525–44. doi: 10.1007/s00068-021-01607-1 PMC7856451 33533957

[14] Vandenbroucke JP von Elm E Altman DG Gotzsche PC Mulrow CD Pocock SJ. Strengthening the Reporting of Observational Studies in Epidemiology (STROBE): explanation and elaboration. Int J Surg. (2014) 12:1500–24. doi: 10.1016/j.ijsu.2014.07.014 25046751

[15] Pfeifer R Pape HC. [Diagnostics and treatment strategies for multiple trauma patients]. Chirurg. (2016) 87:165–73. quiz 174-165. doi: 10.1007/s00104-015-0139-0 26830303

[16] Bouillon B Probst C Maegele M Wafaisade A Helm P Mutschlere M. [Emergency room management of multiple trauma : ATLS(R) and S3 guidelines]. Chirurg. (2013) 84:745–52. doi: 10.1007/s00104-013-2476-1 23979042

[17] Loftis KL Price J Gillich PJ. Evolution of the abbreviated injury scale: 1990-2015. Traffic Inj Prev. (2018) 19:S109–13. doi: 10.1080/15389588.2018.1512747 30543458

[18] Baker SP O'Neill B Haddon W Jr. Long WB. The injury severity score: a method for describing patients with multiple injuries and evaluating emergency care. J Trauma. (1974) 14:187–96. doi: 10.1097/00005373-197403000-00001 4814394

[19] Shankar-Hari M Phillips GS Levy ML Seymour CW Liu VX Deutschman CS. Developing a new definition and assessing new clinical criteria for septic shock: for the third international consensus definitions for sepsis and septic shock (Sepsis-3). JAMA. (2016) 315:775–87. doi: 10.1001/jama.2016.0289 PMC4910392 26903336

[20] Singer M Deutschman CS Seymour CW Shankar-Hari M Annane D Bauer M. The third international consensus definitions for sepsis and septic shock (Sepsis-3). JAMA. (2016) 315:801–10. doi: 10.1001/jama.2016.0287 PMC4968574 26903338

[21] Eguchi A Franz N Kobayashi Y Iwasa M Wagner N Hildebrand F. Circulating extracellular vesicles and their miR "Barcode" Differentiate alcohol drinkers with liver injury and those without liver injury in severe trauma patients. Front Med (Lausanne). (2019) 6:30. doi: 10.3389/fmed.2019.00030 30859103 PMC6397866

[22] Eguchi A Yan R Pan SQ Wu R Kim J Chen Y. Comprehensive characterization of hepatocyte-derived extracellular vesicles identifies direct miRNA-based regulation of hepatic stellate cells and DAMP-based hepatic macrophage IL-1beta and IL-17 upregulation in alcoholic hepatitis mice. J Mol Med (Berl). (2020) 98:1021–34. doi: 10.1007/s00109-020-01926-7 PMC7810220 32556367

[23] Kobayashi Y Eguchi A Tamai Y Fukuda S Tempaku M Izuoka K. Protein composition of circulating extracellular vesicles immediately changed by particular short time of high-intensity interval training exercise. Front Physiol. (2021) 12:693007. doi: 10.3389/fphys.2021.693007 34276412 PMC8280769

[24] Gray WD Mitchell AJ Searles CD. An accurate, precise method for general labeling of extracellular vesicles. MethodsX. (2015) 2:360–7. doi: 10.1016/j.mex.2015.08.002 PMC4589801 26543819

[25] Lund J Saunders CL Edwards D Mant J. Anticoagulation trends in adults aged 65 years and over with atrial fibrillation: a cohort study. Open Heart. (2021) 8:e001737. doi: 10.1101/2021.03.09.21253132 34344724 PMC8336116

[26] Sammy I Lecky F Sutton A Leaviss J O'Cathain A. Factors affecting mortality in older trauma patients-A systematic review and meta-analysis. Injury. (2016) 47:1170–83. doi: 10.1016/j.injury.2016.02.027 27015751

[27] de Knegt C Meylaerts SA Leenen LP. Applicability of the trimodal distribution of trauma deaths in a Level I trauma centre in the Netherlands with a population of mainly blunt trauma. Injury. (2008) 39:993–1000. doi: 10.1016/j.injury.2008.03.033 18656867

[28] Sauaia A Moore FA Moore EE. Postinjury inflammation and organ dysfunction. Crit Care Clin. (2017) 33:167–91. doi: 10.1016/j.ccc.2016.08.006 PMC5129870 27894496

[29] de Vries R Reininga IHF de Graaf MW Heineman E El Moumni M Wendt KW. Older polytrauma: Mortality and complications. Injury. (2019) 50:1440–7. doi: 10.1016/j.injury.2019.06.024 31285055

[30] Halvachizadeh S Baradaran L Cinelli P Pfeifer R Sprengel K Pape HC. How to detect a polytrauma patient at risk of complications: A validation and database analysis of four published scales. PloS One. (2020) 15:e0228082. doi: 10.1371/journal.pone.0228082 31978109 PMC6980592

[31] Capizzi A Woo J Verduzco-Gutierrez M. Traumatic brain injury: an overview of epidemiology, pathophysiology, and medical management. Med Clin North Am. (2020) 104:213–38. doi: 10.1016/j.mcna.2019.11.001 32035565

[32] Galgano M Toshkezi G Qiu X Russell T Chin L Zhao LR. Traumatic brain injury: current treatment strategies and future endeavors. Cell Transplant. (2017) 26:1118–30. doi: 10.1177/0963689717714102 PMC5657730 28933211

[33] Schindler CR Woschek M Vollrath JT Kontradowitz K Lustenberger T Störmann P. miR-142-3p expression is predictive for severe traumatic brain injury (TBI) in trauma patients. Int J Mol Sci. (2020) 21:5381. doi: 10.3390/ijms21155381 32751105 PMC7432828

[34] Crawford AM Yang S Hu P Li Y Lozanova P Scalea T. Concomitant chest trauma and traumatic brain injury, biomarkers correlate with worse outcomes. J Trauma Acute Care Surg. (2019) 87:S146–51. doi: 10.1097/TA.0000000000002256 31246919

[35] Wiles MD. Management of traumatic brain injury: a narrative review of current evidence. Anaesthesia. (2022) 77 Suppl 1:102–12. doi: 10.1111/anae.15608 35001375

[36] Weber B Sturm R Henrich D Lupu L Rottluff K Marzi I. Diagnostic and prognostic potential of exosomal cytokines IL-6 and IL-10 in polytrauma patients. Int J Mol Sci. (2023) 24:11830. doi: 10.3390/ijms241411830 37511589 PMC10380769

[37] Weber B Henrich D Schindler CR Marzi I Leppik L. Release of exosomes in polytraumatized patients: The injury pattern is reflected by the surface epitopes. Front Immunol. (2023) 14:1107150. doi: 10.3389/fimmu.2023.1107150 36969201 PMC10034046

[38] Vedpathak S Sharma A Palkar S Bhatt VR Patil VC Kakrani AL. Platelet derived exosomes disrupt endothelial cell monolayer integrity and enhance vascular inflammation in dengue patients. Front Immunol. (2023) 14:1285162. doi: 10.3389/fimmu.2023.1285162 38235130 PMC10791899

[39] Kumar A Stoica BA Loane DJ Yang M Abulwerdi G Khan N. Microglial-derived microparticles mediate neuroinflammation after traumatic brain injury. J Neuroinflammation. (2017) 14:47. doi: 10.1186/s12974-017-0819-4 28292310 PMC5351060

[40] Park MS Owen BA Ballinger BA Sarr MG Schiller HJ Zietlow SP. Quantification of hypercoagulable state after blunt trauma: microparticle and thrombin generation are increased relative to injury severity, while standard markers are not. Surgery. (2012) 151:831–6. doi: 10.1016/j.surg.2011.12.022 PMC3356502 22316436

[41] Tian Y Salsbery B Wang M Yuan H Yang J Zhao Z. Brain-derived microparticles induce systemic coagulation in a murine model of traumatic brain injury. Blood. (2015) 125:2151–9. doi: 10.1182/blood-2014-09-598805 PMC4375111 25628471

[42] Matijevic N Wang YW Kostousov V Wade CE Vijayan KV Holcomb JB. Decline in platelet microparticles contributes to reduced hemostatic potential of stored plasma. Thromb Res. (2011) 128:35–41. doi: 10.1016/j.thromres.2011.02.011 21421259 PMC3109134

[43] van Manen L Peters AL van der Sluijs PM Nieuwland R van Bruggen R Juffermans NP. Clearance and phenotype of extracellular vesicles after red blood cell transfusion in a human endotoxemia model. Transfus Apher Sci. (2019) 58:508–11. doi: 10.1016/j.transci.2019.05.008 31253560

[44] Straat M Boing AN Tuip-De Boer A Nieuwland R Juffermans NP. Extracellular vesicles from red blood cell products induce a strong pro-inflammatory host response, dependent on both numbers and storage duration. Transfus Med Hemother. (2016) 43:302–5. doi: 10.1159/000442681 PMC5040938 27721707

[45] Hildebrand F Lefering R Andruszkow H Zelle BA Barkatali BM Pape HC. Development of a scoring system based on conventional parameters to assess polytrauma patients: PolyTrauma Grading Score (PTGS). Injury. (2015) 46 Suppl 4:S93–98. doi: 10.1016/S0020-1383(15)30025-5 26542873

[46] Pfeifer R Klingebiel FK Halvachizadeh S Kalbas Y Pape HC. How to Clear Polytrauma Patients for Fracture Fixation: Results of a systematic review of the literature. Injury. (2023) 54:292–317. doi: 10.1016/j.injury.2022.11.008 36404162

[47] Gustafson ML Hollosi S Chumbe JT Samanta D Modak A Bethea A. The effect of ethanol on lactate and base deficit as predictors of morbidity and mortality in trauma. Am J Emerg Med. (2015) 33:607–13. doi: 10.1016/j.ajem.2015.01.030 PMC4453763 25770595

[48] Noren Hooten N Byappanahalli AM Vannoy M Omoniyi V Evans MK. Influences of age, race, and sex on extracellular vesicle characteristics. Theranostics. (2022) 12:4459–76. doi: 10.7150/thno.72676 PMC9169362 35673574

[49] Li Y He X Li Q Lai H Zhang H Hu Z. EV-origin: Enumerating the tissue-cellular origin of circulating extracellular vesicles using exLR profile. Comput Struct Biotechnol J. (2020) 18:2851–9. doi: 10.1016/j.csbj.2020.10.002 PMC7588739 33133426

[50] Kolonics F Szeifert V Timar CI Ligeti E Lorincz AM. The functional heterogeneity of neutrophil-derived extracellular vesicles reflects the status of the parent cell. Cells. (2020) 9:2718. doi: 10.3390/cells9122718 33353087 PMC7766779

[51] Dong X Dong JF Zhang J. Roles and therapeutic potential of different extracellular vesicle subtypes on traumatic brain injury. Cell Commun Signal. (2023) 21:211. doi: 10.1186/s12964-023-01165-6 37596642 PMC10436659

[52] Yang X Chatterjee V Zheng E Reynolds A Ma Y Villalba N. Burn injury-induced extracellular vesicle production and characteristics. Shock. (2022) 57:228–42. doi: 10.1097/SHK.0000000000001938 PMC9246995 35613455

[53] Lian J Zhu X Du J Huang B Zhao F Ma C. Extracellular vesicle-transmitted miR-671-5p alleviates lung inflammation and injury by regulating the AAK1/NF-kappaB axis. Mol Ther. (2023) 31:1365–82. doi: 10.1016/j.ymthe.2023.01.025 PMC10188640 36733250

[54] Matijevic N Wang YW Holcomb JB Kozar R Cardenas JC Wade CE. Microvesicle phenotypes are associated with transfusion requirements and mortality in subjects with severe injuries. J Extracell Vesicles. (2015) 4:29338. doi: 10.3402/jev.v4.29338 26689982 PMC4685295

[55] Liu H Tian Y Xue C Niu Q Chen C Yan X. Analysis of extracellular vesicle DNA at the single-vesicle level by nano-flow cytometry. J Extracell Vesicles. (2022) 11:e12206. doi: 10.1002/jev2.12206 35373518 PMC8977970

