# Supplementary figures and images for: Elevated extracellular particle concentration in plasma predicts in-hospital mortality after severe trauma
Source: Front Immunol. 2024 Jun 12;15:1390380. doi: 10.3389/fimmu.2024.1390380 (PMC11199388; doi:10.3389/fimmu.2024.1390380)

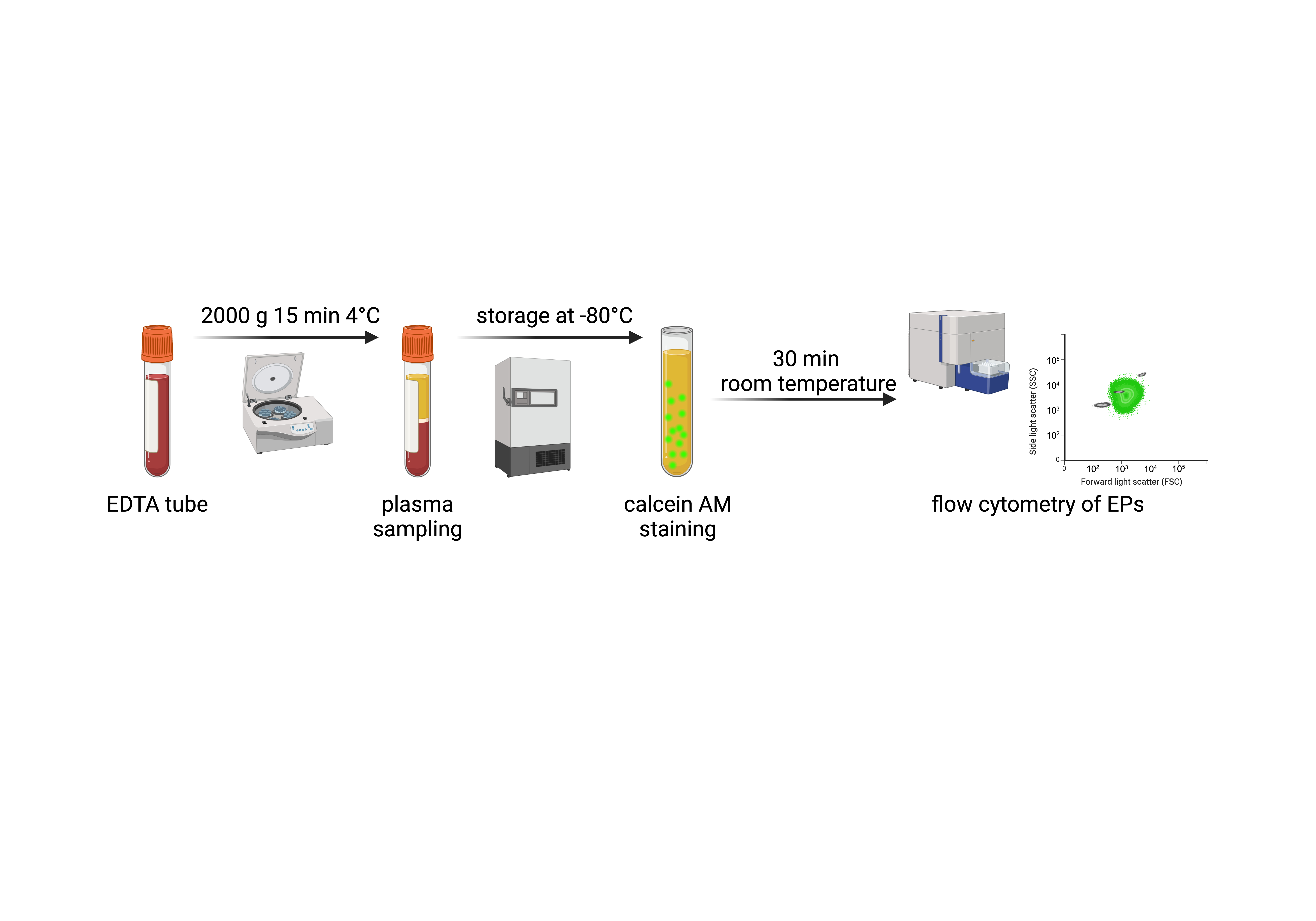

Supplement: Supplementary Figure 1 — Flowchart of the used methodology to assess the extracellular particle analysis. EDTA, Ethylenediaminetetraacetic acid; EPs, extracellular particles. Created with Biorender.com. [file Image_1.tiff]

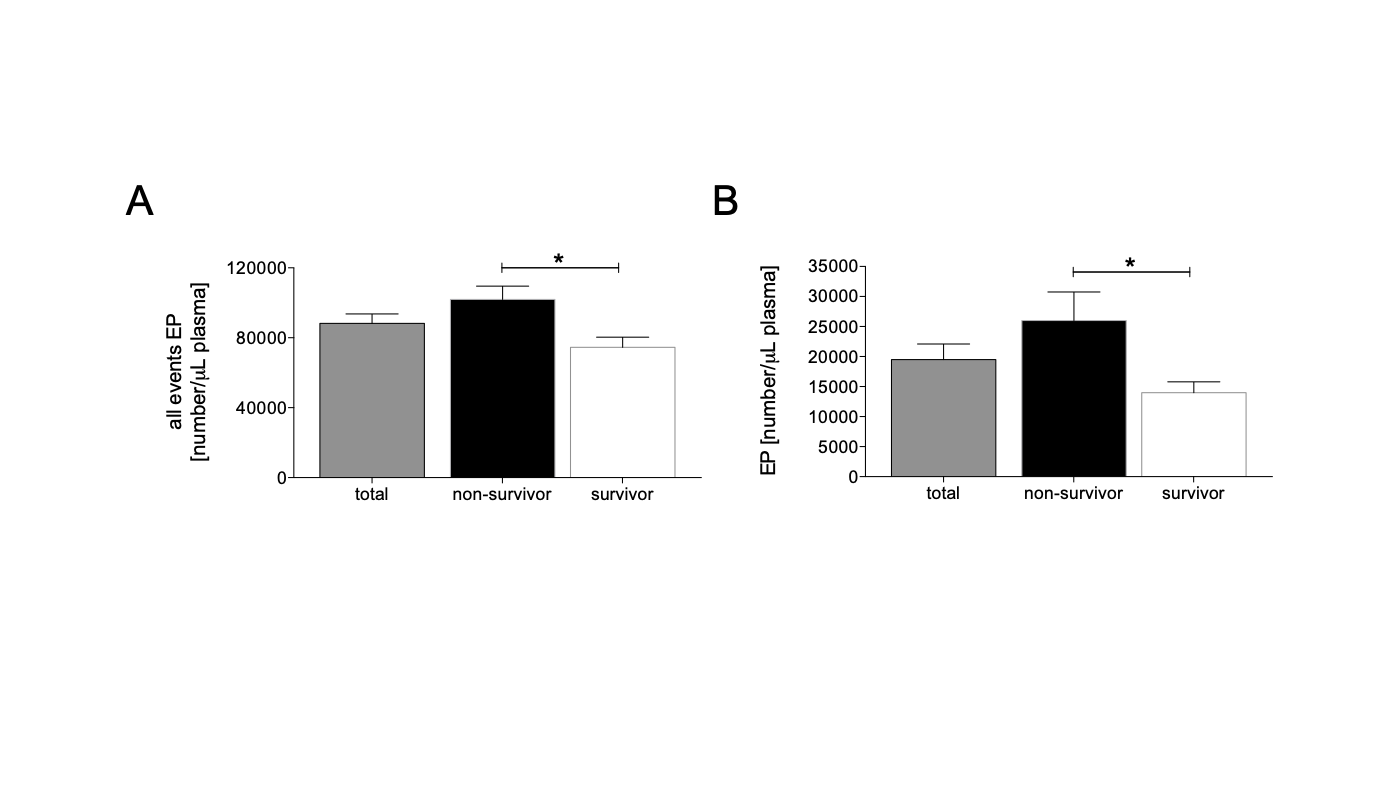

Supplement: Supplementary Figure 2 — Distribution of measured circulating all events (number/µL plasma, A) and EP <200 nm size (number/µL plasma, B) in the matched population containing all matched patients (n=26), non-survivors (n=13) and survivors (n=13). Data are given as mean ± standard error of the mean, *: p <0.05 vs. indicated. [file Image_2.tiff]
