# Supplementary material for: Elevated extracellular particle concentration in plasma predicts in-hospital mortality after severe trauma
Source: Front Immunol. 2024 Jun 12;15:1390380. doi: 10.3389/fimmu.2024.1390380 (PMC11199388; doi:10.3389/fimmu.2024.1390380)
Supplement: Supplementary Table 1 — An overview of patients and injury characteristics and time to the presentation to the emergency department (ED) in the matched-pair population (n=26). Three investigated groups are shown (all matched patients, non-survivors (n=13) and survivors(n=13)). Data are given as mean ± standard error of the mean or median (ISS) with 25% and 75% percentile, p <0.05. AIS, Abbreviated Injury Scale; ISS, Injury Severity Score; n.s., no significance. [file Table_1.docx]

| **trauma severity and mechanism** | **non-survivor**  **(n = 13)** | **survivor**  **(n = 13)** | **p <0.05**  **non-survivor *vs*. survivor** |
| --- | --- | --- | --- |
| **ISS (25% and 75% percentile)** | 25 (21 and 39) | 25 (21 and 39) | n.s |
| **mechanism of injury** |  |  |  |
| **falls, % (n)** | 53.85%, (7) | 46.15%, (6) | n.s |
| **other, % (n)** | 46.15%, (6) | 53.85%, (7) |  |
| **AIS ≥ 3** |  |  |  |
| **head, n, %** | 9, 69.23% | 11, 84.62% | n.s |
| **chest, n, %** | 5, 38.46% | 7, 55.85% | n.s |
| **abdomen, n, %** | 1, 7.69% | 1, 7.69% | n.s |
| **extremity, n, %** | 2, 15.38% | 1, 7.69% | n.s |
| **time to ED presentation (min)** | 66.00 ± 5.15 | 68.18 ± 9.45 | n.s. |
