# Supplementary material for: Elevated extracellular particle concentration in plasma predicts in-hospital mortality after severe trauma
Source: Front Immunol. 2024 Jun 12;15:1390380. doi: 10.3389/fimmu.2024.1390380 (PMC11199388; doi:10.3389/fimmu.2024.1390380)
Supplement: Supplementary Table 2 — Physiologic characteristics and laboratory parameters of the matched-pair population at admission. Three investigated groups are shown [all matched patients (n=26), non-survivors (n=13) and survivors (n=13)]. Data are given as mean ± standard error of the mean, p <0.05. CRP, C-Reactive Protein; ED, Emergency Department; FFP, Fresh Frozen Plasma; IL, Interleukin; INR, International Normalized Ratio; n.s., no significance; PLT, Platelets; PRBC, Packed Red Blood Cells; PTT, activated Partial Thromboplastin Time; SBP, Systolic Blood Pressure; TPT, Thromboplastin Time. [file Table_2.docx]

| **physiological and laboratory measurements** | **non-survivor**  **(n = 13)** | **survivor**  **(n = 13)** | **p <0.05**  **non-survivor *vs*. survivor** |
| --- | --- | --- | --- |
| **SBP, mm Hg** | 127.80 ± 10.17 | 148.30 ± 10.02 | n.s. |
| **shock index (HR/SBP)** | 0.63 ± 0.06 | 0.57 ± 0.07 | n.s. |
| **heart rate** | 74.50 ± 4.93 | 82.85 ± 8.36 | n.s. |
| **breath rate** | 16.00 ± 2.29 | 15.86 ± 2.10 | n.s. |
| **body temperature (°C)** | 35.99 ± 0.38 | 35.70 ± 0.27 | n.s. |
| **PRBC transfusion ED (Units)** | 8.54 ± 5.45 | 1.58 ± 3.34 | n.s. |
| **PRBC transfusion within 24 h (Units)** | 8.70 ± 6.88 | 1.83 ± 0.99 | n.s. |
| **PRBC transfusion total (Units)** | 9.80 ± 6.84 | 2.67 ± 1.07 | n.s. |
| **FFP transfusion within 24 h (Units)** | 8.00 ± 6.28 | 0.00 ± 0.00 | ***yes*** |
| **FFP transfusion total (Units)** | 8.60 ± 6.90 | 0.00 ± 0.00 | ***yes*** |
| **hemoglobin, g/dL** | 11.89 ± 0.64 | 11.36 ± 0.58 | n.s. |
| **TPT (thromboplastin time), %** | 80.92 ± 5.21 | 83.92 ± 6.63 | n.s. |
| **PTT (partial thromboplastin time), (sec)** | 31.42 ± 2.23 | 29.00 ± 1.51 | n.s. |
| **INR** | 1.199 ± 0.067 | 1.275 ± 0.178 | n.s. |
| **fibrinogen, mg/dL** | 196.90 ± 13.87 | 243.40 ± 18.82 | n.s. |
| **PLT count, x 10^3^/µL** | 181.30 ± 18.06 | 224.1 ± 23.48 | n.s. |
| **pH** | 7.265 ± 0.041 | 7.254 ± 0.038 | n.s. |
| **lactate, mg/dL** | 38.00 ± 7.51 | 31.18 ± 5.65 | ***yes*** |
| **leukocytes, U/nL** | 10.34 ± 0.88 | 13.39 ± 1.31 | n.s. |
| **CRP, mg/dL** | 0.33 ± 0.10 | 0.64 ± 0.40 | n.s. |
| **IL-6, pg/mL** | 80.46 ± 25.34 | 222.60 ± 106.10 | n.s. |
| **IL-10, pg/mL** | 19.24 ± 5.03 | 87.37 ± 33.38 | n.s. |
