# Supplementary material for: Elevated extracellular particle concentration in plasma predicts in-hospital mortality after severe trauma
Source: Front Immunol. 2024 Jun 12;15:1390380. doi: 10.3389/fimmu.2024.1390380 (PMC11199388; doi:10.3389/fimmu.2024.1390380)
Supplement: Supplementary Table 3 — Outcome of the matched-pair population (n=26). Three investigated groups are shown [all matched patients (n=26), non-survivors (n=13) and survivors (n=13)]. Data are given as mean ± standard error of the mean, p <0.05. d, day; ICU, Intensive Care Unit; n.s., no significance. [file Table_3.doc]

| **outcome** | **non-survivor**  **(n = 13)** | **survivor**  **(n = 13)** | **p <0.05**  **non-survivor *vs*. survivor** |
| --- | --- | --- | --- |
| **length of ICU stay (days)** | 4.62 ± 1.32 | 11.23 ± 2.37 | ***yes*** |
| **length of hospital stay (days)** | 5.15 ± 1.22 | 17.62 ± 2.40 | ***yes*** |
| **early mortality (<1 day), % (n)** | 23.08%, (3) | - | *early vs. late*  ***yes*** |
| **later mortality (d1-10 days), % (n)** | 76.92%, (10) | - |
| **pneumonia, % (n)** | 15.38%, (2) | 46.15%, (6) | n.s. |
| **sepsis, % (n)** | 7.69%, (1) | 30.77%, (4) | n.s. |
| **septic shock, % (n)** | 7.69%, (1) | 0.00%, (0) | n.s. |
