# Supplementary material for: Elevated extracellular particle concentration in plasma predicts in-hospital mortality after severe trauma
Source: Front Immunol. 2024 Jun 12;15:1390380. doi: 10.3389/fimmu.2024.1390380 (PMC11199388; doi:10.3389/fimmu.2024.1390380)
Supplement: Supplementary Table 4 — Correlation analysis and spearman r values between physiological and laboratory parameters and total extracellular particle (EP) numbers at the emergency department. CRP, C-Reactive Protein; FFP, Fresh Frozen Plasma; INR, International Normalized Ratio; PLT, Platelets; PRBC, Packed Red Blood Cells; SBP, Systolic Blood Pressure. [file Table_4.doc]

| **correlation analyses** | **spearman r** | **p value** |
| --- | --- | --- |
| **age, years** | 0.1787 | 0.0436 |
| **ISS** | -0.01187 | 0.8921 |
| **length of ICU stay, days** | 0.01138 | 0.8977 |
| **length of hospital stay, days** | -0.03063 | 0.7264 |
| **SBP. mm Hg** | 0.2214 | 0.0139 |
| **shock index, HR/SBP** | -0.2411 | 0.0080 |
| **heart rate** | -0.1849 | 0.0366 |
| **breath rate** | 0.001299 | 0.9906 |
| **body temperature, °C** | 0.07376 | 0.4681 |
| **PRBC transfusion ED, Units** | 0.001710 | 0.9850 |
| **PRBC transfusion within 24 h, Units** | 0.08148 | 0.3723 |
| **PRBC transfusion tota, Units** | 0.1070 | 0.2387 |
| **FFP transfusion within 24 h, Unit** | 0.09266 | 0.3100 |
| **FFP transfusion total, Units** | -0.02000 | 0.8262 |
| **hemoglobin, g/dL** | 0.06718 | 0.4423 |
| **TPT (thromboplastin time), %** | -0.1472 | 0.0921 |
| **PTT (partial thromboplastin time), sec** | -0.04507 | 0.6092 |
| **INR** | 0.1614 | 0.0645 |
| **fibrinogen mg/dL** | 0.03049 | 0.7368 |
| **PLT count, x 10^3^/µL** | 0.04104 | 0.6403 |
| **pH** | 0.08396 | 0.3519 |
| **lactate, mg/dL** | -0.01775 | 0.8442 |
| **leukocytes, U/nL** | 0.1741 | 0.0467 |
| **CRP, mg/dL** | -0.01442 | 0.8737 |
